# Combined use of microbubbles of various sizes and single‐transducer dual‐frequency ultrasound for safe and efficient inner ear drug delivery

**DOI:** 10.1002/btm2.10450

**Published:** 2022-11-16

**Authors:** Ai‐Ho Liao, Chih‐Hung Wang, Bo‐Han Wang, Yi‐Chun Lin, Ho‐Chiao Chuang, Hao‐Li Liu, Cheng‐Ping Shih

**Affiliations:** ^1^ Graduate Institute of Biomedical Engineering National Taiwan University of Science and Technology Taipei Taiwan; ^2^ Department of Biomedical Engineering National Defense Medical Center Taipei Taiwan; ^3^ Department of Otolaryngology‐Head and Neck Surgery, Tri‐Service General Hospital National Defense Medical Center Taipei Taiwan; ^4^ Graduate Institute of Medical Sciences National Defense Medical Center Taipei Taiwan; ^5^ Department of Mechanical Engineering National Taipei University of Technology Taipei Taiwan; ^6^ Department of Electrical Engineering National Taiwan University Taipei Taiwan

**Keywords:** cavitation, drug delivery, dual‐frequency ultrasound, hair cell, inner ear, insulin‐like growth factor 1 (IGF‐1), microbubbles, round window membrane

## Abstract

We have previously applied ultrasound (US) with microbubbles (MBs) to enhance inner ear drug delivery, with most experiments conducted using single‐frequency, high‐power density US, and multiple treatments. In the present study, the treatment efficacy was enhanced and safety concerns were addressed using a combination of low‐power‐density, single‐transducer, dual‐frequency US (*I*_SPTA_ = 213 mW/cm^2^) and MBs of different sizes coated with insulin‐like growth factor 1 (IGF‐1). This study is the first to investigate the drug‐coating capacity of human serum albumin (HSA) MBs of different particle sizes and their drug delivery efficiency. The concentration of HSA was adjusted to produce different MB sizes. The drug‐coating efficiency was significantly higher for large‐sized MBs than for smaller MBs. In vitro Franz diffusion experiments showed that the combination of dual‐frequency US and large MB size delivered the most IGF‐1 (24.3 ± 0.47 ng/cm^2^) to the receptor side at the second hour of treatment. In an in vivo guinea pig experiment, the efficiency of IGF‐1 delivery into the inner ear was 15.9 times greater in animals treated with the combination of dual‐frequency US and large MBs (D‐USMB) than in control animals treated with round window soaking (RWS). The IGF‐1 delivery efficiency was 10.15 times greater with the combination of single‐frequency US and large size MBs (S‐USMB) than with RWS. Confocal microscopy of the cochlea showed a stronger distribution of IGF‐1 in the basal turn in the D‐USMB and S‐USMB groups than in the RWS group. In the second and third turns, the D‐USMB group showed the greatest IGF‐1 distribution. Hearing assessments revealed no significant differences among the D‐USMB, S‐USMB, and RWS groups. In conclusion, the combination of single‐transducer dual‐frequency US and suitably sized MBs can significantly reduce US power density while enhancing the delivery of large molecular weight drugs, such as IGF‐1, to the inner ear.

## 1 INTRODUCTION

In recent years, the combined application of therapeutic ultrasound (US) and microbubbles (MBs) has been widely applied to facilitate drug delivery through the blood–brain barrier (BBB) to treat brain diseases,^1^ as a means of local tumor therapy,^2^ transdermal drug delivery,^3^ and inner ear drug delivery.^4^ MBs, which are used as US contrast agents, can also serve as potential drug delivery enhancers and can be combined with acoustic pressure to trigger acoustic cavitation that enhances drug delivery. The acoustic cavitation of MBs can be further classified into stable and inertial cavitation. The nonlinear effect caused by the MB shell is a stable cavitation at low acoustic pressures and an inertial cavitation at high acoustic pressures. However, stable cavitation might not be sufficient for drug delivery, as it is mainly inertial cavitation that leads to pore formation in the cell membrane.^5^ When US‐mediated MBs (USMBs) are applied for inner ear drug delivery, inertial cavitation of MBs generates shock waves on the round window membrane (RWM) leading to pore formation.^6^, ^7^ Stable cavitation from the MB oscillations produces microstreaming that facilitates drug transport through the RWM.^6^

In 2013, our research group was the first to apply USMB cavitation to increase the permeability of the RWM as a way to enhance the efficiency of drug delivery from the middle ear to the inner ear.^6^ The USMB‐mediated delivery of dexamethasone or insulin‐like growth factor 1 (IGF‐1) to the inner ear could successfully result in prevention and therapeutic effects on noise‐induced hearing loss.^8^, ^9^ Subsequent clinical applications replaced US sonication in the middle ear cavity was investigated by placing the single‐frequency US probe in the external auditory canal (EAC) or on the temporal bone.^4^ We also demonstrated that the enhancement of RWM permeability by sonoporation depends on the number of USMB courses, which in turn may be linked to various degrees of outer epithelium injury.^7^ However, exploration of strategies that can reduce the number of US exposures and the US intensity, while maintaining the desired permeability enhancement, is worthwhile to ensure safe clinical applications.

The efficiency of USMBs is determined mainly by the size of MB,^10^, ^11^, ^12^ which acts through resonance frequency, expansion ratio, pressure threshold for inertial cavitation and fragmentation, translational velocity, and a lifetime of stable oscillations.^13^ MBs are tiny bubbles with a gas core, typically between 1 and 10 μm in diameter. They are encapsulated by a shell usually coated with a phospholipid monolayer, proteins, or polymers to reduce surface tension and gas diffusion, thereby stabilizing the gas core. We previously showed that albumin‐shelled MBs of different sizes could be manipulated by adjusting the concentration of albumin or dextrose alone or by combining the albumin and dextrose mixture.^10^ In addition, we found that larger MBs were more resistant to US destruction and helped to enhance the transfection efficiency of auditory hair cells at a constant US power density. Different compositions of phospholipid‐coated MBs had a peak subharmonic response that varied with their size,^14^ suggesting that microstructures in the shell coating may affect the acoustic properties of MBs.

A proposed equation for calculating the theoretical acoustic pressures scattered by nonlinear MB oscillations has also implied that the intensity of acoustic pressure might depend on the MB size.^15^, ^16^ Emmer et al.^16^ used high‐speed optical recordings to investigate the behavior of MBs in a dual‐frequency US field and found that the scattered pressure of USMBs was proportional to the square of the MB radius. A large nonresonant MB may also give a more significant echo than a smaller MB at its resonant frequency,^16^ whereas the eigenfrequency of US is inversely proportional to the equilibrium radius of the MBs.^17^ These theories and findings suggest that a more intense scattered pressure may be obtained with large MBs than with small MBs under a relatively low‐frequency US. The shell viscosity of MB would also be significantly influenced by its radius‐phospholipid coating, thus contributing to a new nonlinear behavior other than the inception of cavitation during USMB treatments.^17^

Several single‐frequency ultrasonic systems are currently developed for combined use with MBs in clinical applications. These systems include ExAblate Neuro 4000 (InSightec, Haifa, Israel); NaviFUS (NaviFUS, Taipei, Taiwan); and SonoCloud‐9 (CarThera, Paris, France).^1^ A dual‐frequency transdermal delivery system has also been proposed, with two US frequencies (20 kHz and 1 MHz) generated separately from various transducers to enhance transdermal delivery efficiency, and it has outperformed a single‐frequency system using 20 kHz.^18^ Different inventions, including intravascular sonothrombolysis,^19^ sonodynamic therapy on melanoma cells,^20^ and the induction of cancer stem cells for differentiation therapy for hepatocellular carcinoma,^21^ have capitalized on the benefits of using dual‐frequency USMBs cavitation instead of single‐frequency US. The critical experimental observation is that multifrequency US can lower the inertial cavitation threshold, thereby improving the power efficiency.^22^ We recently showed that the use of a single‐transducer, dual‐frequency (666 kHz/1 MHz) configuration in combination with USMBs successfully enhanced the growth of hair follicles.^10^ The use of dual‐frequency US caused less skin disruption than was observed with single‐frequency US applied as the FDA‐approved modality, suggesting that dual‐frequency US may be more efficient, effective, and better tolerated than single‐frequency US treatment when applied in vivo.^18^, ^23^

Drug‐loaded MBs can release drugs into target sites when excited by US. This can increase the local drug concentration, while also reducing the unacceptable systemic toxicity, poor distribution, and limited efficacy associated with intravenous administration. Our previous studies have revealed several benefits and potential clinical applications for drug‐loaded MBs and USMB‐related techniques.^6^, ^7^, ^8^, ^9^, ^23^, ^24^, ^25^, ^26^ To enhance the inner ear drug delivery efficacy mediated by USMB, we have demonstrated that the mechanisms underlying the increased permeability of the RWM could involve interruption of the main permeability barrier by disrupting the continuity of the outer epithelial cells caused by acoustic cavitation.^7^ In addition, the enhanced permeation after USMB also involved the transient disruption of the tight junction‐created paracellular barrier in the outer epithelium of the RWM.^26^ However, the EAC is an S‐shaped osseocartilaginous meatus with limited space. Therefore, placing two separate US transducers into the EAC for inner ear drug delivery is essentially not feasible. In the present study, we approached the “Technical Infeasibility” challenge by investigating the efficacy of USMB treatment that used both low‐power‐density (213 mW/cm^2^ *I*_SPTA_) and dual‐frequency (666 kHz/1 MHz) US delivered by a single‐transducer to evaluate the delivery of drugs loaded onto MBs of different diameters. Our aim was to develop a safer and more efficient approach for inner ear drug delivery than is presently available.

## 2 MATERIALS AND METHODS

This research combines in vitro experiments that compare the cavitation effects of different sized MBs and the RWM permeation of IGF‐1 between single‐ and dual‐frequency US and in vivo animal experiments that investigate the cochlear IGF‐1 levels assisted by single and dual‐frequency US. In addition, the safety issues of the cochlea after USMB treatment will be evaluated by measuring auditory brainstem responses and surface preparations of the organ of Corti.

The main methods used are as follows:

### 2.1 Preparation of various diameters of IGF‐1 coated albumin‐shelled MBs

Albumin MBs were prepared as described previously^10^, ^11^ from human serum albumin (HSA) purchased as a sterile 20% solution (Octapharma, Vienna, Austria). MBs of different diameters were prepared by diluting the HSA with physiological saline (pH 7.4, 0.9% sodium chloride) to make stock solutions containing 0.66, 1.32, 2, 3.5, or 5% (w/v) HSA. Briefly, albumin MBs were generated by sonicating a 10 ml mixture of albumin (Table 1) and perfluoropropane (*C*_3_ *F*_8_) gas in physiological saline for 2 min using a sonicator (Branson Ultrasonics, Danbury, CT, USA). The numbers of perfluoropropane‐filled albumin MBs in the solutions were measured with an electrical sensing zone device (MultiSizer III; Beckman Coulter, Fullerton, CA, USA) using a 30 μm aperture probe with measurement boundaries of 0.6–20 μm. The size distribution in the suspension was measured by dynamic light scattering (Zetasizer Nano ZS90; Malvern Instruments, Worcestershire, UK).

**TABLE 1:** The diameter and concentration of albumin‐shelled MBs formed from various concentrations of HSA. Data are means ± *SEM* values

HSA concentration (%)	MBs size (nm)	Number ( × 10^8^ per ml)
0.66	1060.9 ± 41.5	12.2 ± 0.7
1.32	1345.0 ± 50.3	11.6 ± 0.8
2.00	1595.8 ± 67.2	11.6 ± 0.3
3.50	2201.7 ± 71.2	12.3 ± 0.3
5.00	3008.3 ± 56.4	11.7 ± 0.4

Abbreviations: HSA, human serum albumin; MBs, microbubbles.

**FIGURE 1:**
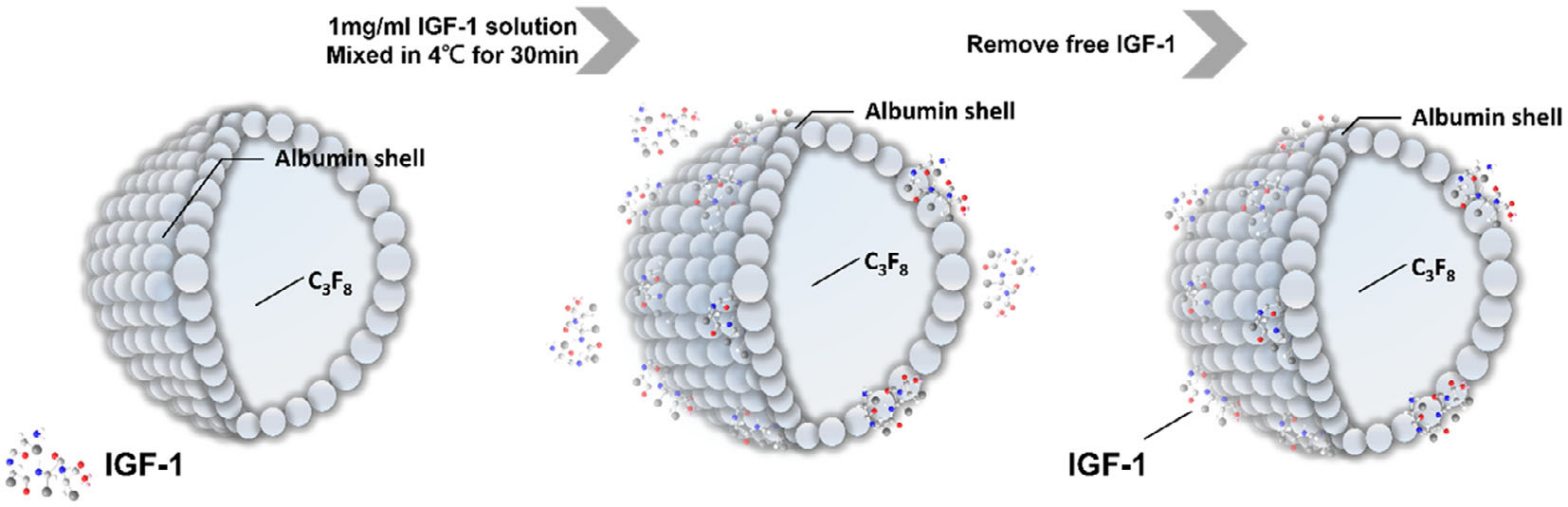
Schematic (not to scale) of the self‐assembly of a positively charged insulin‐like growth factor 1 (IGF‐1) coating onto albumin‐shelled microbubbles (MBs) by electrical adsorption.

Different sizes of self‐assembled albumin‐shelled MBs containing IGF‐1 were prepared with the compositions presented in Figure 1 and Table 1. Albumin contains negative charges, so the surface potential of its shell is less than zero, and the shell can attract positively charged molecules. Human IGF‐1 contains seven positively charged residues in the A‐, C‐, and D‐regions^27^, ^28^; therefore, the IGF‐1 can adhere to albumin‐shelled MBs by electrical adsorption.

The IGF‐1–loaded MBs were produced by mixing 1 mg IGF‐1 in 1 ml Milli‐Q water (18.2 MΩ/cm at 25 °C) and then incubating 0.5 ml of the IGF‐1 solution with 0.5 ml MB solution (original solution of MB production, as shown in Table 1, with concentrations ranging from 11.6 to 12.3 × 10^8^ MBs/ml of each size of MB) on a rotary shaker (50 rpm; Shaker RS‐01, TKS, New Taipei City, Taiwan) for 30 min at 4°C in a *refrigerator*. The MBs were then washed three times to ensure the removal of unbound IGF‐1. The IGF‐MBs in solution were measured with a MultiSizer III device (Beckman Coulter) using a 30‐μm aperture probe and were determined to range in size from 0.6 to 20 μm. The size distribution in the suspension was measured using dynamic light scattering (Nanoparticle Analyzer, Horiba, Kyoto, Japan). Then, 5 μl samples of the MBs and IGF‐MBs were mounted on copper stubs with double‐sided carbon adhesive tape and then coated with platinum (achieved at 0.1 nm/s, 30 mA for 60 s) using an automatic sputter coater (JFC‐1300, JEOL, Tokyo, Japan). The morphologies of the MBs and IGF‐MBs were characterized by high‐resolution field emission scanning electron microscopy (FESEM; FESEM‐6500F, JEOL). The FESEM images were recorded at an accelerating voltage of 15 kV.

### 2.2 Custom‐made single/dual‐frequency US system

**FIGURE 2:**
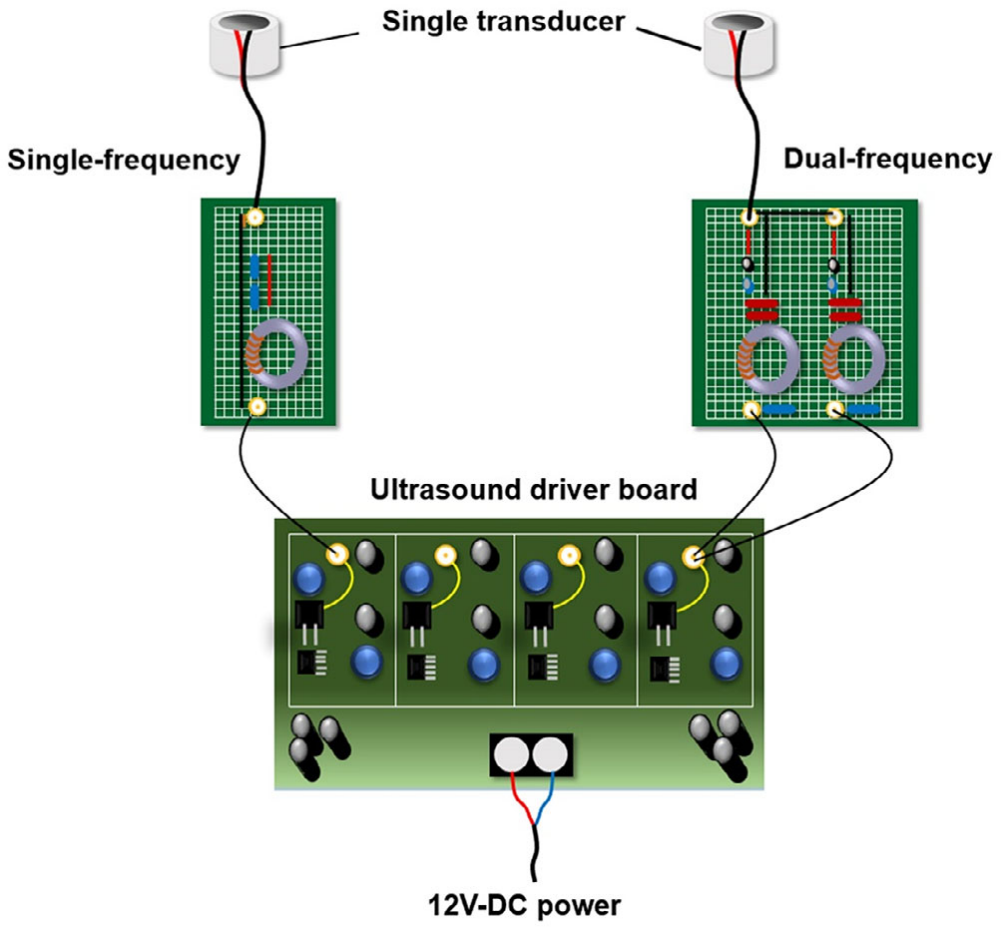
Schematic overview of the custom‐made single ultrasound transducer with a single/dual‐frequency sonication system.

The single/dual‐frequency US system is divided into three parts: a transducer, matching plate, and an US driver board. The single/dual‐frequency US system architecture is shown in Figure 2. The single/dual‐frequency US system is modularized by having the driver circuit board and controlling firmware replace the function generator and RF amplifier for portability and adjustment. This design also avoids the signal reflection that occurs when the impedance is not matched (which affects the life and safety of the system). The characteristic impedance of the circuit for the matching plate was selected as 50 Ω, and a hard piezoelectric (PZT‐4) material was used for the ultrasonic piezo chips in the transducer. The US transducer, PZT‐4 of 25 mm in diameter and 7 mm in thickness, can be switched between single‐frequency (1 MHz) and dual‐frequency (666 kHz + 1 MHz) modes. The operable frequency is measured using a network analyzer (HP 4195A, Keysight Technologies, Santa Rosa, CA, USA), and its two resonance frequencies were determined at 666 kHz and 1 MHz. The piezoelectric chip is fixed into an acrylic package.

The acoustic power was estimated using a UPM‐DT‐10AV force balance (Ohmic Instruments Co., Easton, MD). The transducer was powered by a custom‐designed multiple‐channel driving system with bursts or continuous waves in single‐frequency or dual‐frequency excitation mode. The setting parameters of the system in this research experiment are used with a spatial peak‐pulse average intensity (*I*_SPPA_) of 426 mW/cm^2^, drive burst duty cycle of 50% (*I*_SPTA_ = 213 mW/cm^2^), single frequency of 1 MHz, dual frequency of 666 kHz + 1 MHz, duration time of 3 min, and distance to the target of 1 cm. Using the same measurement method, the *I*_SPTA_ of the commercial US system used in our previous studies (transducer with a diameter of 10 mm, operated at 1 MHz, power density set at 3 W/cm^2^ with a duty cycle of 50% and a pulse repetition period of 250 ms, ST2000V, Nepagene, Ichikawa, Japan) was 655 mW/cm^2^.

### 2.3 Optimization of single/dual‐frequency US parameters for US‐mediated cavitation of MBs of various diameters to enhance drug delivery

The effects of applying single‐ and dual‐frequency US in combination with MBs of various sizes on the RWM were evaluated by high‐frequency US imaging using a commercial animal US imaging system (Prospect, S‐Sharp Corporation, New Taipei City, Taiwan) in tissue‐mimicking agarose phantoms, as described previously.^29^ Real‐time B‐mode high‐frequency US images were obtained using a transducer with a central frequency of 40 MHz to produce axial and lateral resolutions of 30 and 60 μm, respectively (Prospect, S‐Sharp Corporation). The axial and lateral fields of view were 20 and 20 mm. A 2% agarose square column phantom (10 mm × 20 mm × 20 mm) was constructed with a chamber (2 mm × 2 mm × 15 mm) at its center that allowed loading of 250 μl of solution. MBs of various diameters were added (1.2 × 10^7^ MBs/ml), followed by sonication using the single/dual‐frequency US apparatus for 1, 2, 3, or 4 min. Temperatures in the agarose chamber were measured with a thermometer (Optris LS, Optris, Berlin, Germany) before and after sonication.

Our previous work showed that the efficiency of destruction by MBs is proportional to the drug delivery efficacy^4^, ^24^; therefore, the B‐mode images were processed with custom MATLAB programs to evaluate the efficiency of destruction by MBs of various diameters. The destruction efficiency of MBs of various sizes was calculated according to the B‐mode image intensity using the following equation.

(1) $$\text{Efficiency of}\;\mathrm{MBs}\;\text{destruction}\;\left(\%\right)=\frac{I_{0}-I_{n}}{I_{0}}\times 100\%$$

where *I*_0_ is the average pre‐sonication image intensity of the MBs and *I*_*n*_ is the average post‐sonication image intensity.

### 2.4 In vitro RWM permeation by IGF‐1 with single/dual‐frequency US and MBs of various diameters

In vitro RWM penetration was mediated by single/dual‐frequency US and cavitation of MBs of various diameters using static Franz diffusion cells over an area of 2.14 cm^2^ as described previously.^24^ The transducer of the custom‐made single/dual‐frequency US system was positioned 1 cm above an intervening cellulose membrane (MWCO 12–14 kDa; Biomat dialysis membrane, Rainbow Biotechnology Co., LTD., Taipei, Taiwan). The temperature of the diffusion assembly was maintained at 37°C. The transducer of the single/dual‐frequency US and the IGF‐1 MBs of different sizes were applied to the donor compartment (simulated middle ear side), which was completely filled with a receiving medium consisting of 1 ml physiological saline and IGF‐MBs (1.2 × 10^7^ MBs/ml) and occluded with Parafilm (Pechiney Laboratory Safety Products and Apparel, Inc., Chicago, IL, USA). The experimental treatments were MBs of different sizes (1.34 MB: 1345.0 nm MBs, 2.20 MB: 2201.7 nm MBs, and 3.0 MB: 3008.3 nm MBs) and either no US, single‐frequency US (S‐US), or dual‐frequency US (D‐US). The receptor diffusion half‐cell facing the simulated inner ear side was filled with physiological saline (pH 7.4, 6 ml); it contained a stirring bar spinning at 600 rpm during the penetration process. Instead of IGF‐MBs, the solutions in the diffusion cell were filtered through a 0.2 μm (Nalgene, Rochester, NY, USA) or a 0.22 μm (Millex, Darmstadt, Germany) micropore filter. Aliquots (250 μl) of receptor solution were taken at various time intervals (0, 1, 2, 3, and 4 h), and the cell was refilled with the same volume of fresh receptor solution. Samples were kept in a freezer until analysis by ELISA (Epoch, Biotek, Winooski, VT, USA).

### 2.5 Animal study and collection of the perilymph of the inner ear for measurement of IGF‐1 levels

Animals were cared for in compliance with institutional guidelines and regulations (No. IACUC‐21‐083), and all animal experimental protocols were approved by the Institutional Animal Care and Use Committee of the National Defense Medical Center, Taipei, Taiwan. The animal study evaluated the IGF‐1 permeation through the RWM to the cochlea 2 h after USMB using single‐ or dual‐frequency US. The cochlear IGF‐1 distribution and the impact of USMB on hearing were evaluated by examining cochlear surface preparations of the organ of Corti and the auditory brainstem response test, respectively.

Guinea pigs were anesthetized with xylazine (Rompun; Bayer) at 10 mg/kg and ketamine (Imalgene, Merial, Lyon, France) at 40 mg/kg intramuscularly and kept warm with a heating pad. All the surgical procedures were performed with an operating microscope (F‐170, Carl Zeiss, Germany). A retroauricular skin incision was made, as well as a fenestration with a diameter of 4 mm in the tympanic bulla.^11^ The fenestration allowed exposure of the round window and loading of the single/dual‐frequency US transducer and the IGF‐1 MBs.

The groups receiving the single‐frequency US and IGF‐1 MBs (S‐US/IGF‐1 MB) and the groups receiving the dual‐frequency US and IGF‐1 MBs (D‐US/IFG‐1 MB) had the middle ear cavity first filled with IGF‐1 MBs, followed by placement of the transducer on the bony fenestration of the tympanic bulla and a 3 min US sonication. After the US exposure, the IGF‐1 MB solution was preserved in the middle ear cavity. The surgical wound was then closed with sutures. For the round window soaking (RWS) group, the middle ear cavity was filled with IGF‐1 MBs to soak the RWM, but no US treatment was given. The tympanic bulla was dissected and washed with saline. A 10 μl micro‐tip on a pipettor was gently inserted through the RWM into the cochlear duct to aspirate lymphatic fluid. The collected samples were centrifuged immediately at 4°C, 1500 rpm, for 10 min, and then stored at −80°C for later IGF‐1 analysis.

### 2.6 Enzyme‐linked immunosorbent assays of IGF‐1 levels

A commercial competitive human IGF‐1 enzyme‐linked immunosorbent assay (ELISA) kit (R&D System Inc., Minneapolis, MN, USA) was used for IGF‐1 determination. The samples were added to the IGF‐1 ELISA kit well microplate and incubated for 3 h at 2–8°C. The plates were washed with washing buffer to remove the excess liquid. The cold human IGF‐1 conjugate was added, and the plate was incubated 2–8°C for 1 h. The plate was then incubated with the substrate solution at room temperature for 30 min, and the stop solution was added. Colorimetric measurements of IGF‐1 were performed at 450 nm using an ELISA reader (Synergy H4 Hybrid Reader; BioTek Instruments, Winooski, VT). A standard IGF‐1 calibration curve was created to obtain the corresponding concentration of IGF‐1 in the samples on the measured absorption peaks. Five replicate measurements were performed for each concentration of IGF‐1. The minimal detectable IGF‐1 concentration in the samples was 0.1 ng/ml.

### 2.7 Cochlear surface preparation and immunofluorescence analysis of IGF‐1 distribution

After the guinea pigs were euthanized, the tympanic bulla was removed. The cochleae in the tympanic bulla were perfused and fixed with 4% paraformaldehyde for 1 h at room temperature and then decalcified. After a PBS rinse, the tissue surrounding the organ of Corti was removed, and the organ of Corti was carefully preserved. The samples were incubated with Alexa Fluor 488‐conjugated phalloidin (Thermo Fisher Scientific, Eugene, OR, USA) for 30 min at room temperature, rinsed with PBS, mounted in DAPI Fluoromount‐G mounting medium (Southern Biotech, Birmingham, AL, USA), and covered with a coverslip. Fluorescence images were acquired using a confocal laser scanning microscope (Zeiss LSM 880, Carl Zeiss, Jena, Germany). The immunostaining intensity in the images was quantified using ImageJ bundled with 64‐bit Java 1.8.0_172 (https://imagej.nih.gov/ij/download). The staining intensities were expressed in arbitrary units (AU) for the different cochlear turns and subjected to histogram analysis.

### 2.8 Auditory brainstem response recording

The hearing function of the guinea pigs was evaluated by auditory brainstem responses (ABRs), as described previously.^4^, ^24^ For the measurement of ABRs, specific stimuli (clicks and 8, 12, 16, 20, 24, 28, and 32 kHz tone bursts) were produced using SigGen software (Tucker‐Davis Technologies, Gainesville, FL, USA) and were then output monaurally to the EAC via an insert earphone. The average responses from 1024 stimuli, at intensities ranging from 5 to 90 dB sound pressure level for each frequency, were acquired by lowering the sound intensity with 5 dB steps. The ABR threshold was defined as the lowest intensity at which a reproducible deflection in the evoked response trace could be identified.

### 2.9 Statistical analysis

The obtained data were analyzed statistically using the Student's *t* test for comparisons between two groups. Multiple groups were compared using parametric one‐way ANOVA, followed by pairwise multiple comparison procedures or one‐way ANOVA with post hoc Bonferroni correction. A probability value of *p* < 0.05 was considered statistically significant. Data are expressed as the means ± standard error of the mean (*SEM*).

## 3 RESULTS

### 3.1 Characterization of various diameters of albumin‐shelled MBs and IGF‐1‐coated albumin‐shelled MBs

**FIGURE 3:**
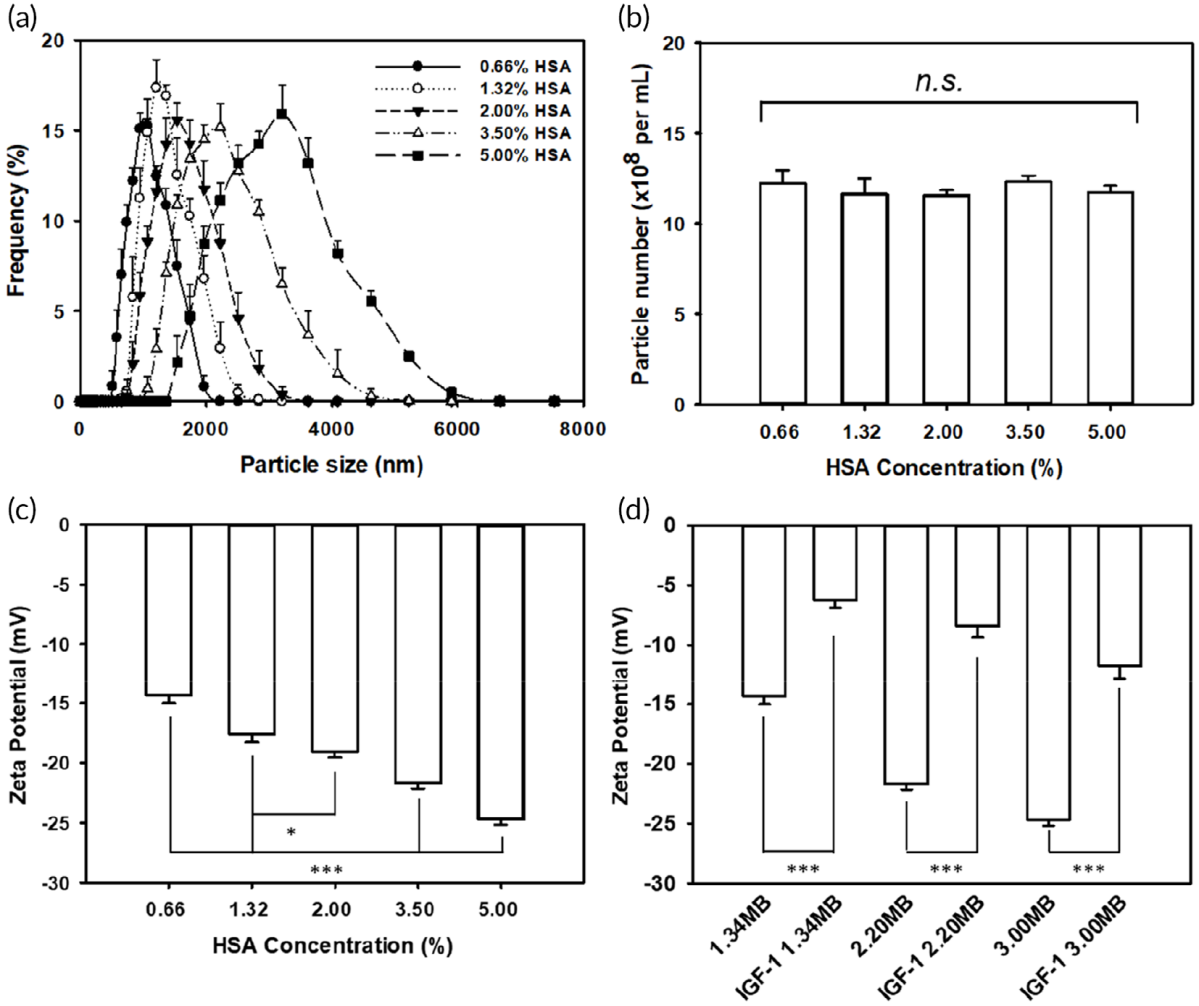
(a) Size, (b) particle distributions, and (c) zeta potentials of microbubbles (MBs) formed from various concentrations of human serum albumin (HSA). (d) Comparison of the zeta potentials of various sized MBs with or without insulin‐like growth factor 1 (IGF‐1) coating. Data are means and *SEM* values. One‐way ANOVA with post hoc Bonferroni test for panel (b) (*p* = 0.895) and panel (c) (*p* < 0.001). One‐tailed Student's *t* test for panel (d) (1.34 MB vs. IGF‐1/1.34 MB, *p* < 0.001; 2.20 MB vs. IGF‐1/2.2 MB, *p* < 0.001; 3.00 MB vs. IGF‐1/3.00 MB, *p* < 0.001). *n*.*s*., no significant difference among all groups; *p < 0.05; ***p < 0.001.

The size of the MBs was influenced by changing the composition ratio of albumin. Albumin compositions in saline of 0.66, 1.32, 2, 3.5, and 5% produced MBs with diameters (means ± *SEM*) of 1060.9 ± 41.5, 1345.0 ± 50.3, 1595.8 ± 67.2, 2201.7 ± 71.2, and 3008.3 ± 56.4 nm, respectively (Figure 3a and Table 1). The concentrations of the produced MBs (ranging from 11.6 to 12.3 × 10^8^ bubbles/ml, *p* > 0.05) were not proportional to the albumin concentration (Figure 3b and Table 1). The zeta potentials of the MBs with compositions of 0.66, 1.32, 2, 3.5, and 5% albumin in Milli‐Q (MQ) water were −14.36 ± 0.7, −17.58 ± 0.7, −19.04 ± 0.5, −21.72 ± 0.4, and −24.68 ± 0.5 mV, respectively, and were proportional to the albumin concentrations (Figure 3c, *n* = 5). We used an ELISA reader to calculate the IGF‐1 adsorption efficiency of MBs, and the results were substituted into the following equation:

(2) $$\text{Adsorption efficiency}\;\left(\%\right)=\frac{\;\text{Adsorption capacity}\;\left(\mathrm{ng}\right)\;}{\text{Total}\;\mathrm{IGF}-1\;\left(\mathrm{ng}\right)}\times 100\%$$

Because the diameters of the 1060.9 ± 41.5, 1595.8 ± 67.2, and 1345.0 ± 50.3 μm MBs were too close to each other, we chose three sizes of MBs (1345.0 ± 50.3, 2201.7 ± 71.2, and 3008.3 ± 56.4 μm) that differed substantially in diameter for the subsequent IGF‐1 adsorption efficiency and IGF‐1 delivery experiments. Table 2 shows that the IGF‐1 loading efficiency of MBs of different sizes (1345.0, 2201.7, and 3008.3 nm) when coated with IGF‐1 (IGF‐1 MBs) was 57.52 ± 2.07, 64.21 ± 1.42, and 71.11 ± 1.52%, respectively, and the zeta potential was proportional to the MB diameter (Figure 3d). The zeta potential was also significantly more positive for the IGF‐1 MBs than for the uncoated MBs.

**TABLE 2:** The IGF‐1 adsorption efficiency of human serum albumin MBs with various diameters. Data are means ± *SEM* values

MB size (nm)	Total IGF‐1 (ng)	Adsorption capacity (ng)	Adsorption efficiency (%)
1345.0 ± 50.3	500	287.59 ± 17.62	57.52 ± 3.52%
2201.7 ± 71.2	500	328.47 ± 17.62	65.69 ± 3.52%
3008.3 ± 56.4	500	355.54 ± 11.89	71.11 ± 2.38%

Abbreviations: IGF‐1, insulin‐like growth factor 1; MBs, microbubbles.

**FIGURE 4:**
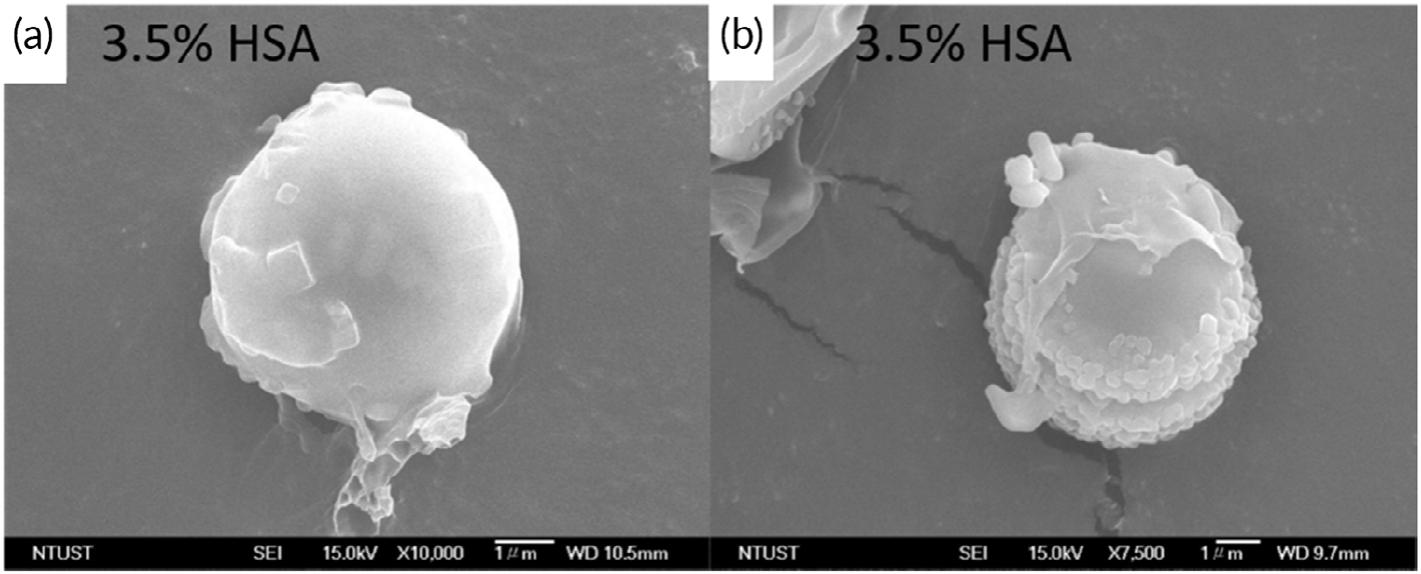
Scanning electron micrographs of the albumin‐shelled microbubbles (MBs) (a), and insulin‐like growth factor 1 (IGF‐1) MBs (b)

The amount of adsorbed IGF‐1 increased with increasing MB size (Table 2), further confirming a size‐dependent IGF‐1 adsorption. Large MBs with a high surface area and negative charge were able to attract greater amounts of IGF‐1. Adsorption of positively charged IGF‐1 by the MBs decreases the number of negative charges on the albumin‐shelled MBs. In this study, an electrical adsorption method was used to enable adsorption of the positively charged IGF‐1 onto the negatively charged MB surface. The TEM images demonstrated that, in general, the MB surface was relatively smooth, whereas an uneven particulate complex was present on the MB surface after the adsorption of IGF‐1 (Figure 4).

### 3.2 High‐frequency US imaging for optimizing single/dual‐frequency US sonication

**FIGURE 5:**
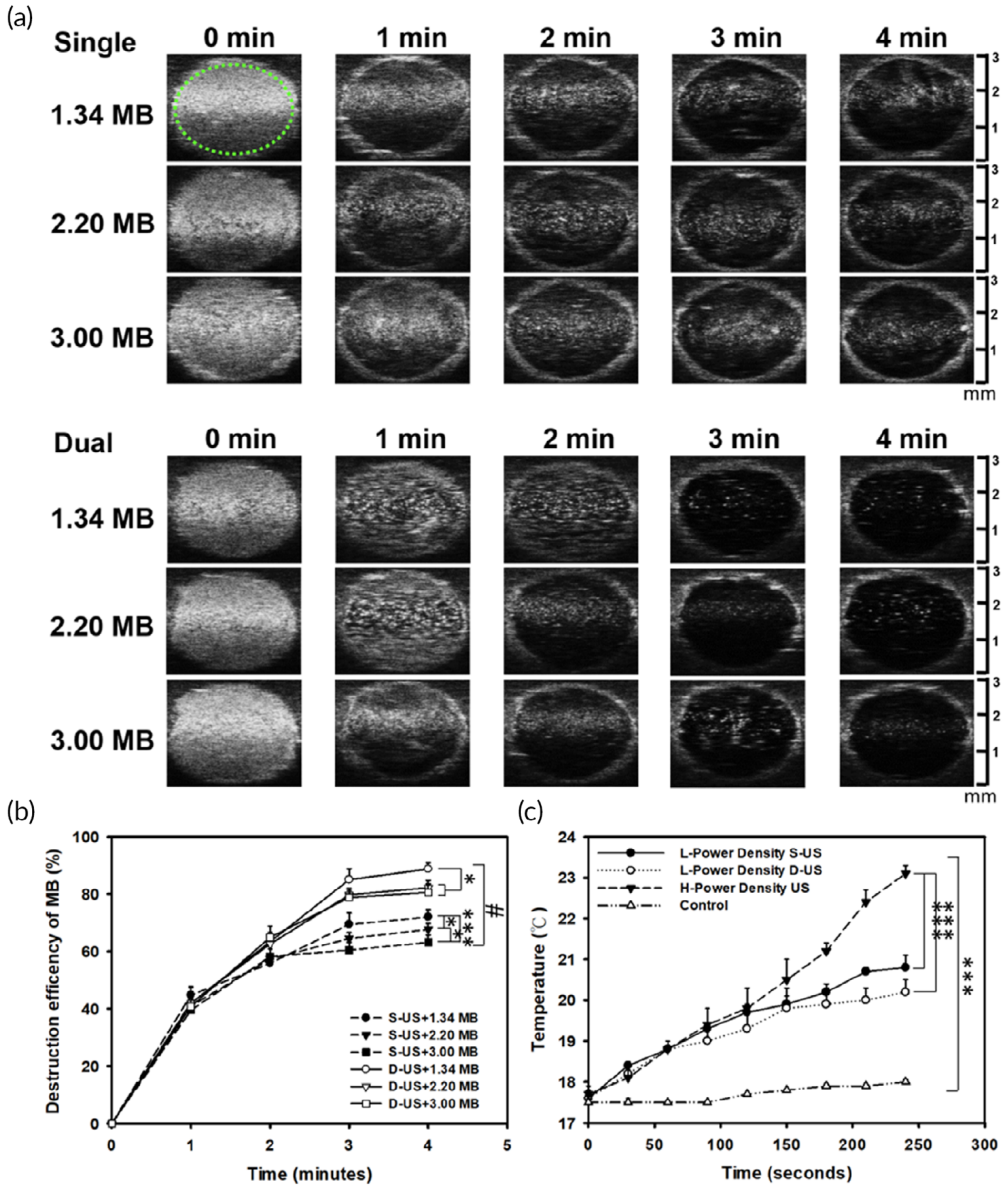
In vitro high frequency ultrasound (US) images of microbubbles (MBs) of various diameters before (0 min) and after single‐ or dual‐frequency US sonication for 1, 2, 3, and 4 min. (a) Images representing the MBs in the agarose phantoms. The region of interest (green dotted‐line circle) was drawn over the entire MB‐loaded chamber. (b) Quantification of the destruction efficiency in (a) (*n* = 5 per group). # Indicates a significant difference (*p* < 0.001) for the destruction efficiency of the three different MB sizes groups between single‐ and dual‐frequency US sonication. (c) Temperature changes after L‐Power (low‐power, *I*_SPTA_ = 213 mW/cm^2^) density, single‐frequency ultrasound (S‐US), dual‐frequency ultrasound (D‐US) frequency, and H‐Power (high‐power, *I*_SPTA_ = 655 mW/cm^2^) density US (single‐frequency) insonation at various time points within the agarose chamber. (*p < 0.05, ***p < 0.001) Data are means and *SEM* values. Fisher LSD multiple comparison in a one‐way ANOVA for panel (b) (S‐US + 1.34 MB vs. S‐US + 2.20 MB, *p* = 0.036; S‐US + 2.20 MB vs. S‐US + 3.00 MB, *p* = 0.018; D‐US + 1.34 MB vs. D‐US + 2.20 MB, *p* = 0.015; D‐US + 1.34 MB vs. D‐US + 3.00 MB, *p* = 0.017; D‐US + 2.20 MB vs. D‐US + 3.00 MB, *p* = 0.937). One‐way ANOVA with post hoc Bonferroni test for panel (c) (H‐power density US vs. L‐power density S‐US, *p* < 0.001; H‐power density US vs. L‐power density D‐US, *p* < 0.001; L‐power density S‐US vs. L‐power density D‐US, *p* = 0.396). *p < 0.05; ***p < 0.001.

The differences between single‐frequency and dual‐frequency US for cavitation of MBs of various sizes were determined using the small animal US imaging system (Prospect, S‐Sharp Corporation) to record and analyze the MB B‐mode images in the phantom (a circle chamber). The B‐mode images of MBs would reduce the brightness intensity over time during US sonication for 1–4 min, as shown in Figure 5a. The results of pairwise comparisons of the destruction efficiency of the three different MB sizes using single/dual‐frequency US (1.34 μm: 69.49 ± 4.0% vs. 85.1 ± 3.55%; 2.20 μm: 64.5 ± 2.1% vs. 79.7 ± 2.2%; 3.00 μm: 60.5 ± 2.8% vs. 78.8 ± 2.4%) (Figure 5b) revealed a higher efficiency for dual‐frequency US than for single‐frequency US regardless of the MB size (*p* < 0.001). Smaller MBs also tended to be more substantially destroyed compared to larger ones. Our previous studies revealed that most of the MBs are destroyed to complete the cavitation effect when the destruction efficiency reached more than 80%.^23^ The percentage of MB destruction efficiency is the consequence of inertial cavitation. And Figure 5c shows that the S‐US group (lower power density and single‐frequency US, *I*_SPTA_ = 213 mW/cm^2^), D‐US group (lower power density and dual‐frequency US, *I*_SPTA_ = 213 mW/cm^2^), and US group (higher power density and single‐frequency US, *I*_SPTA_ = 655 mW/cm^2^, ST2000V, Nepagene, Ichikawa, Japan) increased in temperature by 2.6, 2.3, and 3.5°C, respectively, after sonication for 180 s; by 3.2, 2.6, and 5.4°C, respectively, after sonication for 240 s, suggesting that a higher power density and use of single‐frequency US could result a significantly higher temperature rise than observed with S‐US or D‐US. The destruction efficiencies would reach a plateau after sonication for 3 min either in single or dual‐frequency US. Therefore, we conducted the subsequent experiments with the selected sonication time of 3 min.

### 3.3 In vitro permeation of IGF‐1 under single/dual‐frequency US mediated the cavitation of MBs of various diameters

**FIGURE 6:**
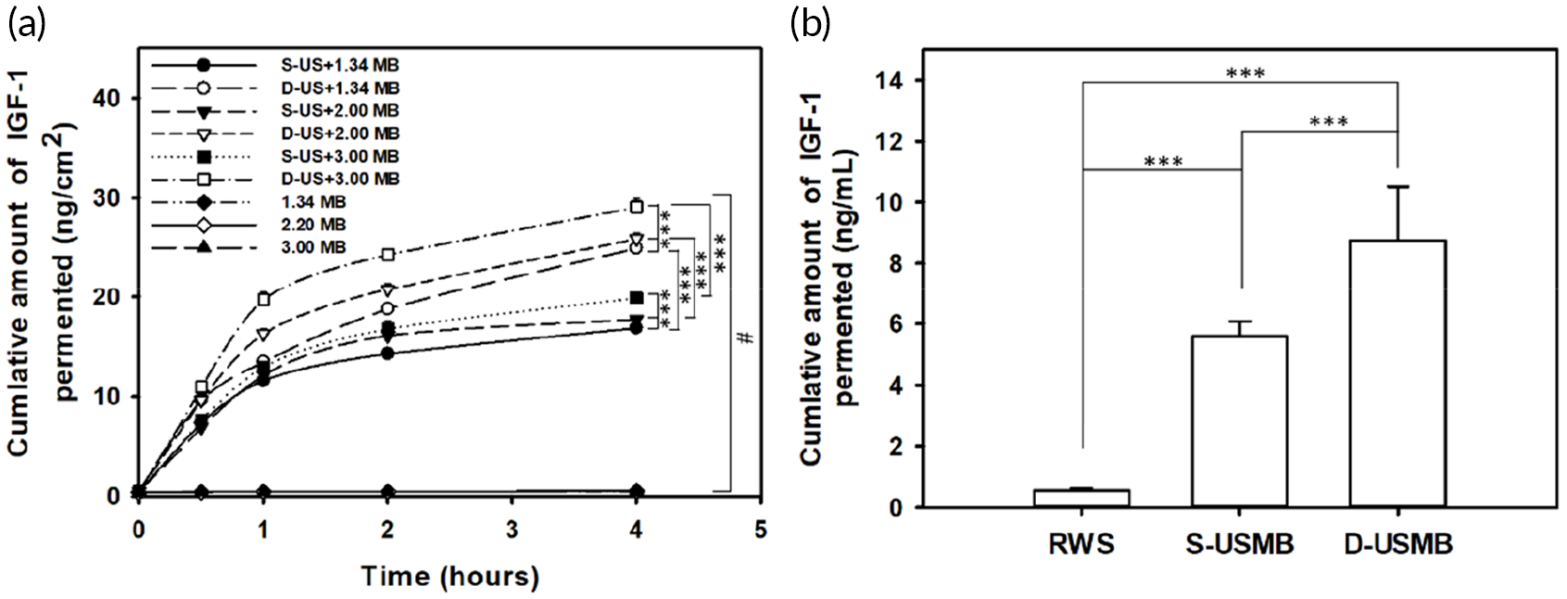
(a) Comparison of in vitro insulin‐like growth factor 1 (IGF‐1) penetration through a cellulose membrane in a Franz diffusion cell for IGF‐1–loaded microbubbles with diameters of 1.34, 2.20, and 3.00 μm with or without single‐ or dual‐frequency ultrasound. (b) Comparison of in vivo IGF‐1 concentrations in the cochlear perilymphatic fluid of guinea pigs after single‐frequency or dual‐frequency ultrasound of 3.00 μm microbubbles (MBs). The results are expressed as the mean ± standard error of the mean, with *n* = 6 for each bar. RWS, round window membrane soaking with 3.00 μm MBs; S‐USMB, single‐frequency ultrasound combined with 3.00 μm MBs; D‐USMB, dual‐frequency ultrasound combined with 3.00 μm MBs. ***p < 0.001. # Indicates a significant difference (*p* < 0.001) between MB groups with or without single/dual‐frequency ultrasound. Holm–Sidak multiple comparison in a one‐way ANOVA for panel (a) (*p* < 0.001). One‐way ANOVA with post hoc Bonferroni test for panel (b) (*p* < 0.001); ***p < 0.001.

Figure 6a shows the IGF‐1 penetration through the cellulose membrane over 4 h in a Franz diffusion cell in groups treated with MBs of different sizes with or without single or dual US. At 2 h, the IGF‐1 concentrations were higher in the dual‐frequency groups than in the single‐frequency groups for MBs of each size (18.8 ± 0.29 ng/cm^2^ in D‐US + 1.34 MB vs. 14.3 ± 0.29 ng/cm^2^ in S‐US + 1.34 MB, 20.8 ± 0.61 ng/cm^2^ in D‐US + 2.20 MB vs. 16.1 ± 0.47 ng/cm^2^ in S‐US + 2.20 MB cm^2^, and 24.3 ± 0.47 ng/cm^2^ in D‐US + 3.00 MB vs. 16.8 ± 0.79 ng/cm^2^ in S‐US + 3.00 MB). The IGF‐1 concentrations in each control group (soaking in IGF‐1 and 1.34, 2.20, and 3.0 MB) were around 0.5 ± 0.09 ng/cm^2^. At 4 h, the cumulative amount of IGF‐1 was significantly higher in the D‐US groups than in the S‐DS groups (*p* < 0.001). Of these D‐US treatments, 3.0 MBs exhibited the best IGF‐1 penetration and release performance.

### 3.4 Single/dual‐frequency US‐mediated cavitation of 3.00 μm MBs promotes drug delivery to the inner ear without incurring auditory damage

The results from the Franz diffusion cell experiments led to the adoption of IGF‐1 coated 3.00 μm MBs combined with single/dual‐frequency US to test the differences in IGF‐1 penetrating through the RWM in the animal experiments. The D‐USMB group had the highest cochlear IGF‐1 concentration (8.75 ± 1.76 ng/ml), followed by the S‐USMB group (5.58 ± 0.48 ng/ml), and then the control RWS group (0.55 ± 0.08 ng/ml) (Figure 6b). Compared with the control RWS group, the D‐USMB treatment was considerably more efficient than the S‐USMB treatment (*p* < 0.005 vs. *p* < 0.05) in delivering IGF‐1 into the cochlea. The drug delivery efficiency into the inner ear was increased by up to 60% by dual‐frequency versus single‐frequency US treatment.

**FIGURE 7:**
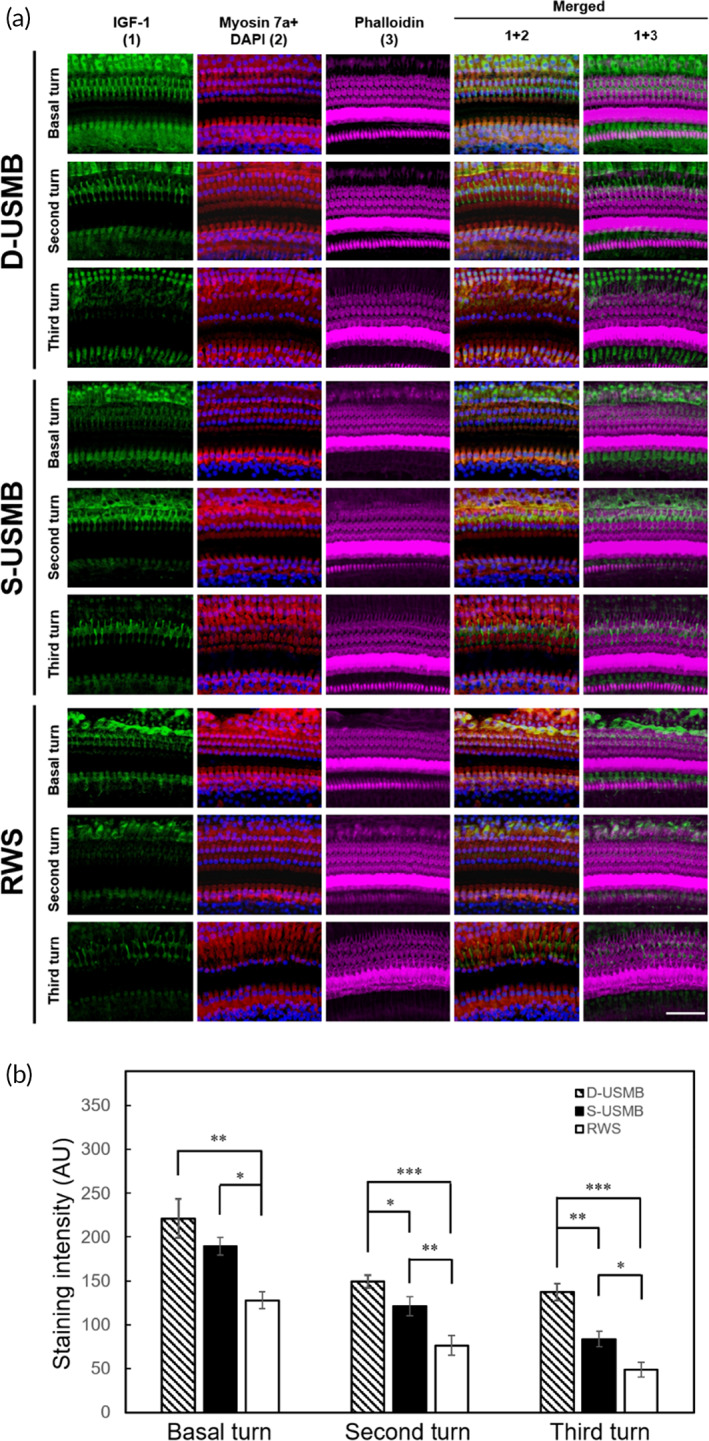
Dual‐frequency ultrasound microbubble (D‐USMB) and single‐frequency USMB (S‐USMB) treatments enhance insulin‐like growth factor 1 (IGF‐1) diffusion into the cochlea. Samples were obtained from the cochleae 1 day after IGF‐1 treatments. (a) Representative confocal microscopy images of the basal turn after immunofluorescence staining show a stronger distribution of IGF‐1 (green) in the D‐USMB and S‐USMB groups than in the RWS group. In the second and third turns, the D‐USMB group had the strongest IGF‐1 distribution among the three groups. Six replicate experiments were conducted. Myosin 7a(a) (red); phalloidin (magenta); DAPI (blue). (b) Histogram representations of the mean fluorescence intensity of IGF‐1 staining. Data are shown as the mean ± *SEM* (*n* = 6 for each bar). Scale bar: 50 μm. D‐USMB, dual‐frequency ultrasound microbubble treatment; S‐USMB, single‐frequency ultrasound microbubble treatment; RWS, round window soaking. One‐way ANOVA with post hoc Bonferroni test for panel (b) (Basal turn: D‐USMB vs. RWS, *p* = 0.0014; D‐USMB vs. S‐USMB, *p* = 0.3266; S‐USMB vs. RWS, *p* = 0.0273. Second turn: D‐USMB vs. RWS, *p* < 0.001; D‐USMB vs. S‐USMB, *p* = 0.0247, S‐USMB vs. RWS, *p* = 0.0012. Third turn: D‐USMB vs. RWS, *p* < 0.001; D‐USMB vs. S‐USMB, *p* = 0.00135; S‐USMB vs. RWS, *p* = 0.034). *p < 0.05; **p < 0.005; ***p < 0.001

The confocal microscopy analysis of the cochlea also revealed a more robust distribution of IGF‐1 in the basal turn in the D‐USMB and S‐USMB groups than in the RWS group (Figure 7; D‐USMB vs. RWS, *p* = 0.001; S‐USMB vs. RWS, *p* = 0.021). In the second turn of the cochlea, the D‐USMB group had the most robust IGF‐1 distribution compared to the other two groups (D‐USMB vs. S‐USMB, *p* = 0.025; D‐USMB vs. RWS, *p* < 0.001). In the third turn of the cochlea, the D‐USMB group also presented the most robust IGF‐1 distribution compared to the other two groups (D‐USMB vs. S‐USMB, *p* = 0.002; D‐USMB vs. RWS, *p* < 0.001).

Furthermore, in the region with inner hair cells, the D‐USMB group showed the most intense IGF‐1 labeling fluorescence among the three groups. Overall, the use of either single‐frequency or dual‐frequency USMBs could facilitate IGF‐1 delivery to the inner ear. More importantly, a more efficient inner ear drug delivery was obtained with dual‐frequency US‐mediated cavitation than with single‐frequency US, as well as a more substantial IGF‐1 distribution in the organ of Corti.

**FIGURE 8:**
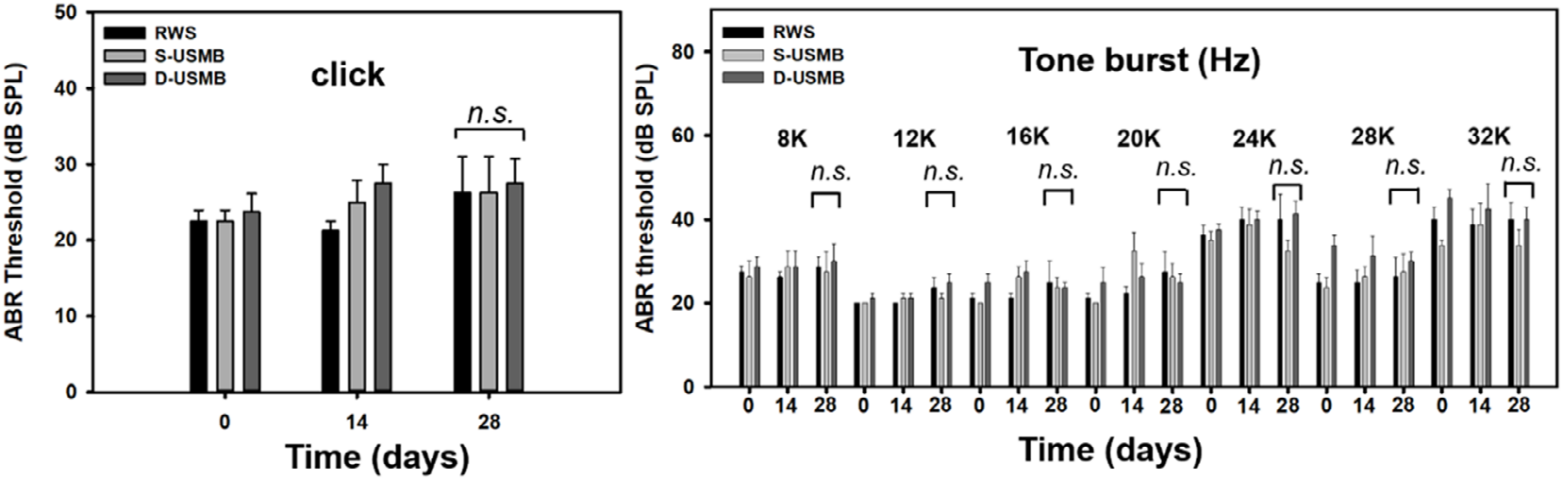
Hearing evaluations of guinea pigs after dual‐frequency ultrasound microbubble (D‐USMB) and single‐frequency USMB (S‐USMB) treatments. The auditory brainstem response (ABR) threshold recording with click and tone burst stimuli before (day 0) and at a 4‐week follow‐up in the D‐USMB, S‐USMB, and round window soaking (RWS) groups. The results are expressed as the mean ± *SEM*, with *n* = 4 for each bar. One‐way ANOVA (click stimuli, *p* = 0.972; tone burst stimuli, *p* = 0.381). *n*.*s*., no significant difference among all groups

**FIGURE 9:**
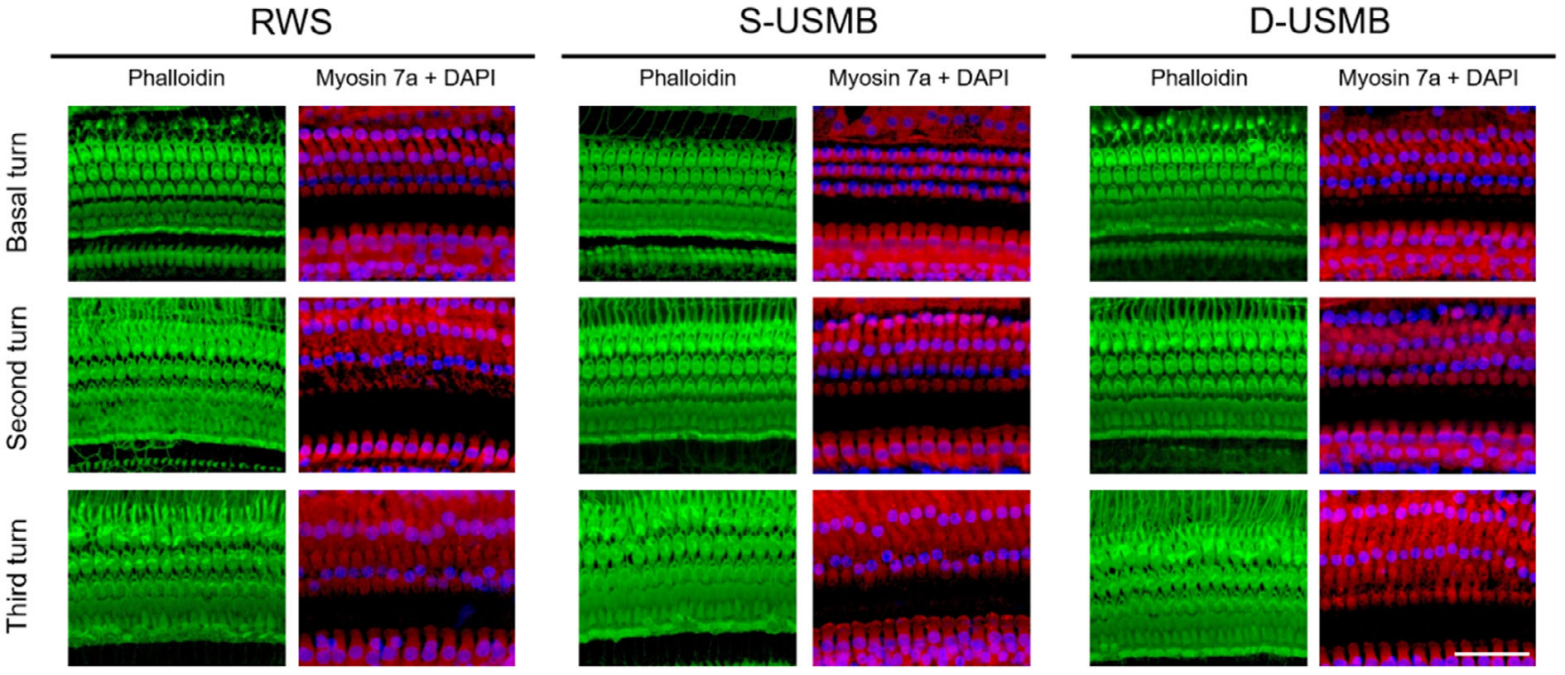
Representative images of confocal microscopy analysis of cochlear surface preparations obtained at 4 weeks after dual‐frequency ultrasound microbubble (D‐USMB) and single‐frequency USMB (S‐USMB) treatments. No cochlear hair cell loss was observed in the D‐USMB, S‐USMB, or round window soaking (RWS) groups. The staining shows the nuclei (blue, DAPI), filamentous actin (green, phalloidin), and hair cells (red, myosin 7a). Four replicates of these experiments were conducted. Scale bar: 50 μm

Functional evaluation of hearing showed no significant difference in the ABR threshold before and 4 weeks after US‐mediated treatment with 3.00 μm MBs in the S‐USMB and D‐USMB groups, and no differences in ABR thresholds among the S‐USMB, D‐USMB, and RWS groups (Figure 8; with click stimuli, *p* = 0.972; with tone burst stimuli, *p* = 0.381). The hair cell loss in the organ of Corti at 4 weeks after single‐frequency or double‐frequency mediated cavitation of 3.00 μm MBs in the cochlea (Figure 9) revealed no significant hair cell loss in all turns of the cochlea. These findings suggest that the cavitation of 3.00 μm MBs mediated by single‐frequency or dual‐frequency US does not cause either structural or functional damage to the inner ear.

## 4 DISCUSSION

Local drug delivery to the inner ear has none of the drawbacks of the systemic route and can be applied via intratympanic or intracochlear approaches^30^, ^31^; however, the selective permeability of the RWM may limit the efficiency of intratympanic drug delivery, especially for high‐molecular‐weight drugs. The present study demonstrated that larger albumin MBs carried high molecular weight protein like IGF‐1 (~7600 Da) and when assisted by dual‐frequency (*I*_SPTA_ = 213 mW/cm^2^), USMBs can provide a 15.9‐fold increase in the delivery efficiency compared to spontaneous RWM absorption, and is 1.57 times more efficient than single‐frequency USMB. This suggests that dual‐frequency US can provide a safe advantage by using low US power density to promote the inner ear drug delivery. IGF‐1 is regarded as a new, promising therapeutic agent for treating sensorineural hearing loss and has undergone clinical trials in patients with idiopathic sudden sensorineural hearing loss that is refractory to systemic steroid therapy.^32^, ^33^ Dual‐frequency US‐mediated IGF‐1 MBs therefore have high potential for future clinical application.

Previous studies reported in the literature have mostly investigated the preparation techniques for optimizing the formulation size, stability, and drug‐loading capabilities of lipid MBs.^34^ The echogenicity, stability, vascular flow, sonoporation behavior, and the consequent therapeutic use and performance were strongly affected by MB size.^35^, ^36^ According to theory, large‐sized MBs, rather than small‐sized MBs, are also expected to be more therapeutically efficient for drug delivery due to their more desirable sonoporation capability and higher drug‐loading capacity (the optimal particle size is around 2.3 to 2.9 μm for gene transfer at 1 MHz).^37^, ^38^ In the present study, the adsorption efficiency of IGF‐1 onto HSA MBs of various diameters indicated that the larger surface area and the higher degree of negative charge of large‐diameter MBs can promote the electrical adsorption of greater amounts of IGF‐1.

Our previous study evaluated the feasibility of laboratory production of MBs of different sizes for use with single‐frequency US as a gene transfection method in auditory cells. The efficiency of gene transfer increased with increasing MB diameter, and the related gene transfection efficiency was facilitated using different‐sized MBs in auditory cells.^10^ However, some previous studies have proposed using cationic lipid MBs to augment the interactions between MBs and cells and to reduce the separation of plasmid DNA from MBs to facilitate gene transfer efficiency.^39^ Furthermore, since albumin has negative charges, the positively charged IGF‐1 can be adsorbed onto albumin‐shelled MBs by electrical adsorption, thereby aiding a strong interaction with the negatively charged lipid bilayers of the cell membrane and enhancing the drug delivery effects.

We previously also found that the US intensity sufficient to mediate MBs destruction (*I*_SPTA_ = 655 mW/cm^2^) was proportional to the drug delivery efficiency. Under a lower US power density (*I*_SPTA_ = 213 mW/cm^2^) for 3 min and using either single‐ or dual‐frequency US, the destruction efficiency was significantly higher for 1.34 μm MBs than for the larger 2.20 and 3.00 μm MBs. However, the drug delivery efficacy increased dramatically for the larger 3.00 μm compared to the other two types of MBs, especially after dual‐frequency US. The larger MBs are believed to be more therapeutically efficient than the smaller ones because of their desirable sonoporation nature and high drug‐loading capacity.^15^ The scattered pressure of USMBs is also proportional to the square of the MB radius, and the eigenfrequency of US is inversely proportional to the MB diameter.^16^ Therefore, using a lower US frequency of 666 kHz in the D‐USMB group could result in more scattered pressure and enhanced drug delivery on larger MBs.

The choice to shift the second resonance frequency from 20 kHz in previous studies to 666 kHz in the present study was a consequence of the presence of MBs. Solely driving US to generate bubble cavities requires high intensity, and the bubble dimension can be arbitrarily expanded up to 100 μm in size, with the resonance better match around 20–60 kHz, and distinct from seeded MBs with a smaller size of 10 μm have better resonance around 600–1000 kHz.^40^ As shown in previous study utilizing frequency combination of 20 kHz and 1 MHz,^18^ the use of a dual‐frequency US system without the involvement of MBs would require a relatively high US exposure level to trigger “intrinsic” cavitation. By contrast, the use of MBs 1–3 μm in diameter in our current USMB system better matches the resonance at 666 kHz, and with the MBs involvement, low power intensity (213 mW/cm^2^ ISPTA) is sufficient to trigger cavitation, thereby meeting the need for a safer and more efficient approach for inner ear drug delivery.

The combination of US and MBs has been shown to be the most promising approach for achieving localized BBB permeation for delivering drugs into the brain parenchyma without damaging the surrounding tissue.^41^, ^42^, ^43^ Previous studies have indicated that the volume of medication administered to brain tissue by USMBs was strongly dependent on both the US power density and the MB diameter.^12^, ^44^ Larger diameter bubbles and lower power density were determined to be safe and consistent in their associated BBB opening. The interaction between larger MBs and US could induce BBB opening through nonlinear oscillation and does not cause inertial cavitation.^12^, ^44^ Therefore, in our present study, inertial cavitation (MB destruction) induced by lower US power density is not proportional to drug delivery efficiency.

Compared with our previous inner ear drug treatment parameters, dual‐frequency US can reduce the power density and reduce the number of treatments, but the treatment duration is extended from 1 to 3 min each time. A treatment extension of 3 min increased the treatment area temperature following exposure to single/dual‐frequency US by 2.6–3.2°C. Compared with the previously used high power density, single‐frequency US, which increased the temperature by 1.1°/min, application of these lower power density single/dual‐frequency US for 3 min only slightly increased the temperature by 1.5–2.1°C.

## 5 CONCLUSION

This study is the first to investigate the drug‐coating capacity of HSA MBs of different particle sizes and their effects on drug delivery efficiency. The larger surface area and the higher degree of negative charges in large‐diameter MBs caused the adsorption of greater amounts of IGF‐1. The interaction between large MBs and dual‐frequency US could increase IGF‐1 delivery through nonlinear MB oscillations with partial inertial cavitation. Therefore, inertial cavitation (MB destruction) capability is not proportional to drug delivery efficiency. The combined use of dual‐frequency US (666 kHz + 1 MHz) and 3.0 μm MBs can enhance the delivery of drugs, including IGF‐1, and other large molecular weight agents, into the inner ear while reducing the damaging effects and required power density of US.

## AUTHOR CONTRIBUTIONS

**Ai‐Ho Liao:** Conceptualization (equal); funding acquisition (equal); project administration (equal); supervision (equal); writing – original draft (equal). **Chih‐Hung Wang:** Conceptualization (equal); funding acquisition (equal); supervision (equal); writing – original draft (equal); writing – review and editing (equal). **Bo‐Han Wang:** Data curation (equal); formal analysis (equal); project administration (equal); software (equal). **Yi‐Chun Lin:** Data curation (equal); project administration (equal); validation (equal); visualization (equal). **Ho‐Chiao Chuang:** Investigation (equal); methodology (equal); validation (equal). **Hao‐Li Liu:** Methodology (equal); validation (equal). **Cheng‐Ping Shih:** Conceptualization (equal); data curation (equal); funding acquisition (equal); investigation (equal); methodology (equal); project administration (equal); supervision (equal); writing – review and editing (lead).

## CONFLICT OF INTEREST

The authors have declared that no conflict of interest exists.

### PEER REVIEW

The peer review history for this article is available at https://publons.com/publon/10.1002/btm2.10450.

## Data Availability

The main data supporting the findings of this study are available within the paper. The associated raw data are available from the corresponding author on reasonable request.
